# Amyloidogenic and non-amyloidogenic transthyretin variants interact differently with human cardiomyocytes: insights into early events of non-fibrillar tissue damage

**DOI:** 10.1042/BSR20140155

**Published:** 2015-01-14

**Authors:** Pallavi Manral, Natàlia Reixach

**Affiliations:** *Department of Molecular and Experimental Medicine, The Scripps Research Institute, 10550 North Torrey Pines Road, La Jolla, CA 92037, U.S.A.

**Keywords:** amyloidosis, cardiomyocytes, cytotoxicity, protein misfolding, proteotoxicity, transthyretin, BCA, bicinchoninic acid, DHE, dihydroethidium, FAC, familial amyloid cardiomyopathy, FAP, familial amyloid polyneuropathy, HBSS, Hank’s balanced salt solution, LC–ESI–MS, liquid chromatography–electrospray ionization mass spectrometry, MBCD, methyl-β-cyclodextrin, NSB, non-specific binding, PI, protease inhibitor, RAP, receptor-associated protein, ROS, reactive oxygen species, SSA, senile systemic amyloidosis, TCEP, tris-(2-carboxyethyl)phosphine, T_4_, thyroxine, TTR, transthyretin, WT, wild-type

## Abstract

TTR (transthyretin) amyloidoses are diseases characterized by the aggregation and extracellular deposition of the normally soluble plasma protein TTR. *Ex vivo* and tissue culture studies suggest that tissue damage precedes TTR fibril deposition, indicating that early events in the amyloidogenic cascade have an impact on disease development. We used a human cardiomyocyte tissue culture model system to define these events. We previously described that the amyloidogenic V122I TTR variant is cytotoxic to human cardiac cells, whereas the naturally occurring, stable and non-amyloidogenic T119M TTR variant is not. We show that most of the V122I TTR interacting with the cells is extracellular and this interaction is mediated by a membrane protein(s). In contrast, most of the non-amyloidogenic T119M TTR associated with the cells is intracellular where it undergoes lysosomal degradation. The TTR internalization process is highly dependent on membrane cholesterol content. Using a fluorescent labelled V122I TTR variant that has the same aggregation and cytotoxic potential as the native V122I TTR, we determined that its association with human cardiomyocytes is saturable with a K_D_ near 650 nM. Only amyloidogenic V122I TTR compete with fluorescent V122I for cell-binding sites. Finally, incubation of the human cardiomyocytes with V122I TTR but not with T119M TTR, generates superoxide species and activates caspase 3/7. In summary, our results show that the interaction of the amyloidogenic V122I TTR is distinct from that of a non-amyloidogenic TTR variant and is characterized by its retention at the cell membrane, where it initiates the cytotoxic cascade.

## Abstract 2

The differential interaction and processing of an aggregation-prone, disease-associated transthyretin variant with human cardiac cells, compared with a non-disease associated transthyretin mutant may explain the basis of the early events of tissue damage in the transthyretin amyloidoses.

## INTRODUCTION

The amyloidoses are a subset of protein misfolding diseases characterized by the deposition of β-sheet rich protein aggregates resulting in organ dysfunction and death [1]. One of the more than 30 human proteins associated with amyloid deposition is TTR (transthyretin). Physiological TTR is a homotetramer mainly synthesized in the liver and choroid plexus of the brain, which circulates in plasma and CSF (cerebrospinal fluid) serving as carrier of T_4_ (thyroxine). In plasma, TTR is also known to be a transporter of retinol bound to retinol-binding protein. It is thought that TTR might have other functions such as that of being a general detoxifier of unwanted metabolites, including Aβ (amyloid β-peptide) [2].

The TTR amyloidoses are systemic disorders of protein deposition. SSA (senile systemic amyloidosis) and FAC (familial amyloid cardiomyopathy) are syndromes characterized by cardiac deposition of WT (wild-type) and mutant TTR variants, respectively. FAP (familial amyloid polyneuropathy) is characterized by dominant peripheral nerve and cardiac mutant TTR deposition. More than 100 TTR variants have been identified and are related to amyloid deposition. Several other tissues are also affected by WT or mutant TTR deposition, including lungs, gut, carpal tunnel, skin and kidneys. It is not clear why some TTR variants tend to deposit more in some tissues than in others [2]. The most common TTR mutation worldwide is V122I which is found in 4% of African–Americans and produces late onset FAC, resulting in congestive heart failure and death [3,4].

Biophysical studies have demonstrated that the stability of the native tetrameric TTR governs its amyloidogenic potential [5,6]. The dissociation of the homotetramer into its corresponding monomers is the rate-limiting step in the amyloidogenic cascade, which is followed by the misfolding and aggregation of the monomer subunits resulting in amorphous aggregates and amyloid fibrils [7].

For many years it was thought that the mass of deposited amyloid fibrils was solely responsible for tissue dysfunction. However, markers of tissue damage characteristic of inflammation, apoptosis and ROS (reactive oxygen species) generation have been found in tissues of human and transgenic mouse models carriers of mutant TTR variants, well before amyloid deposits can be detected [8–10]. These markers have been associated with amorphous TTR aggregates that can be observed in the early stages of FAP evolution. More recently, it has been shown that hearts of 3 months old transgenic mice overexpressing human WT TTR had a significant increase in the transcription levels of genes related to inflammation and immune response, compared to non-transgenic littermates [11]. These transgenic animals do not display cardiac TTR deposition in the form of amorphous aggregates or amyloid fibrils until 18 months of age [10]. Moreover, in tissue culture systems using cells derived from organs that are targets for TTR deposition, recombinant amyloidogenic TTR variants reduce cell viability and activate caspase-3, whereas the stable and non-amyloidogenic TTR variant T119M does not have such effects [12,13]. In these systems, monomers or small oligomers generated during the amyloidogenic cascade, and not large soluble or insoluble amorphous aggregates or amyloid fibrils, appear to be responsible for the cytotoxicity [12]. Thus the data suggest that amyloidogenic TTR-induced tissue damage may precede TTR fibril deposition.

We have previously demonstrated that AC16 human cardiomyocytes are sensitive to several amyloidogenic TTR variants but not to the stable and non-amyloidogenic T119M TTR [13]. AC16 cells are derived from adult human cardiomyocytes of the ventricle, the site of TTR deposition in SSA and FAC, and display many markers of primary cardiomyocytes [14]. We noted that WT TTR in this system was not cytotoxic when the recombinant protein was purified at room temperature, but it was cytotoxic when the protein was purified at 4°C. This behaviour was also noted in a tissue culture system using human neuroblastoma cells [15]. We also showed that in AC16 cells amyloidogenic TTR-induced cytotoxicity can be prevented by kinetically stabilizing the native tetramer with small molecules that bind in the TTR T_4_ binding pocket preventing its dissociation and the ensuing amyloidogenic cascade. We have now used this FAC tissue culture model to understand how amyloidogenic (V122I TTR) and non-amyloidogenic (T119M TTR) variants interact with the human cardiomyocytes. The stark differences found in the interaction modes of these two proteins with the cells point to an explanation for the amyloidogenic V122I TTR cytotoxic properties. Our data have implications for the design of new therapeutic strategies to prevent the earliest events producing tissue damage in the TTR amyloidoses. The findings might also be relevant to the interaction of other amyloidogenic proteins with their cell targets and provide a broader understanding of the mechanisms of tissue damage related to protein misfolding, and the ensuing gain of toxic function responsible for these diseases.

## EXPERIMENTAL

### TTR preparation and purification

Recombinant TTR was prepared and purified in an *Escherichia coli* expression system as described elsewhere [12]. The last step of purification consisted in gel filtration chromatography on a Superdex 75 column (GE Biosciences) to obtain tetrameric TTR free of aggregates. When the recombinant TTR was intended to be used for biophysical studies, the gel filtration purification was performed in 10 mM phosphate buffer (sodium) pH 7.6/100 mM KCl/1 mM EDTA buffer (GF buffer); when the TTR was intended for cell culture experiments, HBSS (Hank's balanced salt solution; Mediatech) buffer was used instead. The plasmids to obtain the TTR variants C10A/V122I/P125C and C10A/V122I/E127C were produced by PCR-assisted site directed mutagenesis using the V122I TTR plasmids as template. The new plasmids were sequenced to ensure that the desired mutations had been introduced. All the purified recombinant proteins were stored at −80°C at concentrations lower than 2.5 mg/ml, conditions under which the proteins are stable and do not aggregate. LC–ESI–MS (liquid chromatography–electrospray ionization mass spectrometry) was used to confirm the molecular mass of the recombinant proteins: V122I TTR, 13905.4 (expected, 13906.6), T119M, 13921.6 (expected 13922.6), C10A/V122I/P125C, 13878.9 (expected 13880.5), C10A/V122I/E127C, 13847.5 (expected 13848.5).

### Labelling of V122I TTR variants with fluorescent probes

The cysteine residues of V122I TTR, C10A/V122I/E127C TTR and C10A/V122I/P125C TTR variants were labelled with Oregon Green 488 maleimide (O-6034, Molecular Probes) using thiol chemistry. The cysteine residues of C10A/V122I/E127C TTR and C10A/V122I/P125C TTR variants were also derivatized with Alexa Fluor 488 C5-maleimide (A-10254, Molecular Probes) following the manufacturer's instructions. Briefly, TTR solutions (~2 mg/ml) were dialysed against 50 mM of sodium phosphate buffer pH 7.2 with 100 μM TCEP [tris(2-carboxyethyl) phosphine-hydrochloride, Biosynth], at room temperature for 2 h. TCEP was required to maintain the cysteine residues in reduced form and available for derivatization. Stock solutions of the fluorophores were prepared at 5 mM (in DMSO) and added dropwise to TTR solutions with vigorous agitation. We used 5× and 8× molar excess dye:TTR for Alexa Fluor 488 and Oregon Green 488, respectively. The conjugation reactions were allowed to proceed at 4°C overnight in the dark, under mild agitation. In all the subsequent steps the labelled proteins were protected from the light. The crude reaction mixtures were dialysed against GF buffer at room temperature for 2 h and the proteins re-purified by gel filtration at 4°C on a Superdex 75 column (GE Biosciences) in GF buffer to remove aggregates that may have formed during the labelling process. LC–ESI–MS was used to confirm the nature of the derivatized proteins and the efficiency of the procedure. The molecular mass of the labelled proteins were: C10A/V122I/P125C-Oregon Green 488, 14343.8 (expected, 14343.5), C10A/V122I/E127C-Oregon Green 488, 14311.1 (expected, 14311.5), C10A/V122I/P125C-Alexa Fluor 488, 14577.9 (expected, 14577.5), C10A/V122I/E127C-Alexa Fluor 488, 14545.8 (expected, 14545.5). The degree of labelling was 2.5–2.8 TTR subunits per TTR tetramer for the Oregon Green 488-labelled proteins and four TTR subunits per TTR tetramer for the Alexa Fluor 488-labelled proteins.

### Covalent V122I kinetic stabilization with a resveratrol analogue

V122I TTR was kinetically stabilized with a resveratrol analogue (SM) that binds covalently to Lys^15^ of TTR in the T_4_-binding pocket (compound 4 in [16]). V122I TTR (5 μM) was incubated with 10 μM of SM at 25°C overnight. The solution was then dialysed in HBSS and concentrated. We measured the degree of covalent binding by LC–ESI–MS. Two peaks were observed in the spectrogram at 1:0.7 ratios corresponding to modified:unmodified V122I subunits, respectively. The measured molecular mass of the covalently modified polypeptide was 14155.33 (expected, 14156).

### Acid-mediated TTR aggregation and fibril formation

pH-dependent fibril formation studies were performed as described previously [17,18]. Briefly, TTR solutions (8 μM) in GF buffer containing 100 μM TCEP were diluted 1:1 with acetate buffer (200 mM sodium acetate/100 mM KCl/1 mM EDTA, pH 4.2) to achieve a final pH of 4.4. The mixtures were incubated at 37°C for 3 days in cluster tubes (Genesee Scientific) or in Eppendorf tubes without agitation.

### Measurement of amounts of soluble and insoluble TTR

Samples of 400 μl of aggregated TTR solutions in Eppendorf tubes were centrifuged at 20000 ***g*** for 30 min at 4°C. The protein concentration of the supernatants was measured using 1/2 area 96-well UV-transparent plates in triplicate (50 μl/well). To determine the amount of insoluble (aggregated) TTR in the recovered precipitates, 200 μl of an 8 M GndCl solution was added. The samples were then vortexed briefly and left at room temperature for 5 min to allow for the disassembly of TTR aggregates to take place. The TTR concentration was then measured by UV spectrophotometry in ½ area, 96-well UV-transparent plates in triplicate, using 8 M GndCl as blank. The percentages of protein in the supernatant and the pellet were calculated from the total initial soluble protein content at time zero. The experiments were done at least twice in triplicate.

### Cell culture

The human cardiomyocyte cells AC16 [14] were grown in 10 cm tissue culture dishes in DMEM (Dulbecco's modified Eagle's medium):F12 (1:1) (Mediatech) supplemented with 10% FBS, 1 mM Hepes, 2 mM L-glutamine, 100 units/ml penicillin, 100 μg/ml streptomycin in a 5% (v/v) CO_2_ incubator at 37°C. The cells were passaged 2–3 times/week at a split ratio of 1:10 to 1:20.

### Cell viability assays

AC16 cells (70–90% confluent) were seeded in black-wall clear-bottom 96 well plates (50 μl, 300 cells/well) in Opti-MEM (Invitrogen), supplemented with 5% (v/v) FBS, 2 mM L-glutamine, 100 units/ml penicillin, 100 μg/ml streptomycin, 1 mM Hepes and 45 mM CaCl_2_ (Opti-MEM seeding medium) and incubated overnight at 37°C in a 5% CO_2_ atmosphere. The next day 50 μl/well of the appropriate TTR solutions or vehicle (HBSS) were added to the wells. The cells were incubated for 24 h at 37°C and cell viability was measured using the resazurin reduction assay [13]. Briefly, 10 μl/well of resazurin (500 μM in PBS) were added to the cells and incubated for 2–3 h at 37°C. The fluorescence resulting from the reduction of resazurin to resorufin by metabolically active cells was measured on a Tecan Safire2 multiplate reader (Tecan) using excitation/emission wavelengths of 530/590 nm with 10 nm bandwidth. Cell viability was calculated as the percentage of fluorescence of TTR-treated cells with respect to the control cells (vehicle-treated cells) after subtraction of the blank (wells with no cells). Averages and the S.E.M. are presented. The experiments in which MBCD (methyl-β-cyclodextrin) was used, the seeded cells were pre-incubated with 2–16 mM MBCD (Sigma-Aldrich) solubilized in DMSO, or DMSO only as vehicle control, for 15 min at 37°C. The rest of the steps were performed as above. In these experiments, the final concentration of TTR was 8 μM and the final concentrations of MBCD ranged from 1 to 8 mM.

### Saturation experiments

The human cardiomyocyte AC16 cells (400000 cells/sample) were incubated on ice with C10A/V122I/P125C-Alexa Fluor 488 TTR (0–4 μM solutions in HBSS), named henceforth f-V122I TTR, in the absence or presence of 100 molar equivalents of unlabelled V122I TTR. The TTR solutions were then removed and the cells washed twice with 500 μl cold HBSS and subsequently lysed in RIPA buffer [50 mM Tris–HCl (pH 7.5)/150 mM NaCl/0.1% (w/v) SDS/1% (v/v) Triton X-100/0.5% (w/v) sodium deoxycholate] in the presence of Complete™, a protease inhibitor cocktail (Roche), henceforth named RIPA-PI (protease inhibitor), and subjected to two cycles of freeze/thawing (−80°C/37°C). Fluorescence of the cell lysates was measured at Exc/Em 488/515 nm using a 10 nm bandwidth. Fluorescence intensity values were normalized to total protein content in the samples measured by BCA (bicinchoninic acid) assay. The fluorescence intensity values obtained from the samples incubated with 100× molar excess of unlabelled V122I TTR was considered NSB (non-specific binding) and subtracted from the samples incubated with f-V122I TTR to obtain specific binding. Each experiment consisted of biological duplicates. The specific binding data fit best to one site binding (hyperbola) curve (Graph Prism). The experiment was repeated four times. The data presented in Figure 2 correspond to one representative experiment.

### Competition experiments

The AC16 human cardiomyocytes (400000 cells/sample) were incubated for 3 h on ice with f-V122I TTR (320 nM) in the absence or presence of increasing concentrations of unlabelled V122I or T119M TTR (0-3.2 μM). The TTR solutions were then removed and the cells were washed and lysed as above. The fluorescence intensity associated with the cell lysates was measured and the values were normalized by total protein measured by BCA. The normalized values were plotted against the logarithm of the unlabelled TTR concentration in nM units. The data were fit by a sigmoidal curve of variable slope with Graph Prism. Each experiment consisted of biological duplicates. The experiments were repeated six times. The data presented in Figure 3 correspond to one representative experiment. The V122I concentration that displaced 50% of f-V122I TTR (IC_50_) was calculated from the average for the six experiments ± S.D. The dissociation constant (Ki) for V122I TTR was calculated from the Cheng–Prusoff equation [*K_i_*=IC_50_/(1+*D*/*K_D_*)] [19], where *D* is the concentration of f-V122I TTR and K_D_ is the equilibrium constant for the f-V122I TTR determined in the previous section.

### Quantification of TTR associated with human cardiomyocytes

AC16 cells (1.6×10^6^ cells/experimental point) were incubated with V122I TTR or T119M TTR in HBSS containing 0.1% BSA at 4°C or at 37°C for 3 h. In some experiments the cells were first pre-incubated with MBCD or DMSO (vehicle) for 4 h before the addition of the proteins or HBSS. After incubation with TTR, the cells were spun at 1000 ***g*** for 5 min at 4°C and the supernates were removed by aspiration. The cells were then washed twice with cold 5 mM EDTA in PBS. Half of the samples were then incubated with 0.125% (w/v) trypsin in PBS (2 ml) for 10 min at 4°C, whereas the other half were incubated in PBS only. Subsequently, the cells were spun at 1000 ***g*** for 5 min at 4°C and the supernates were removed by aspiration. After two more cycles of washing (in PBS), spin and aspiration, the cells were lysed with 200 μl RIPA-PI. The samples were sonicated for 3 min, 3 s pulses of amplitude 20 with 1 s rest, and spun at 5000 ***g*** for 5 min at 4°C to remove cell debris. The samples were analysed in 15% acrylamide/bisacrylamide SDS gels by electrophoresis (SDS–PAGE) and Western blot developing with a rabbit anti-TTR antibody (1:1,000 in TBST, Dako A0002) followed by a goat anti-rabbit IgG-HRP (horse radish peroxidase) (1:5,000 in TBST, Thermo Scientific PI31460) and West-Pico developing agents (Thermo scientific). X-ray films were exposed and developed appropriately to avoid saturated bands, and scanned at high resolution (600 dpi). The band intensities were quantified using ImageJ (NIH 1.48v) according to the gel analysis protocol described in http://rsb.info.nih.gov/ij/docs/menus/analyze.html#gels. Briefly, the images were converted to 8-bit greyscale format. Profile plots for every band were obtained and the area enclosed in these plots was measured. Profile plots corresponding to blank areas were also measured. These blank areas were subtracted from the TTR bands to obtain background-subtracted density areas. Each Western blot contained two biological duplicates. Averages and S.D. of the calculated areas were plotted. The experiments were repeated at least three times. Data from one representative experiment are shown.

The experiments in which proteasome (MG132) and lysosome acidification (NH_4_Cl) inhibitors were used, the cells, seeded in 24-well plates for 24 h before treatment, were incubated with 0.5 μM MG132, 10 mM NH_4_Cl or vehicle only for 30 min prior to the addition of TTR (4 μM final concentration). The concentrations of MG132 and NH_4_Cl were selected as those producing minimal cytotoxicity to the cells as measured by resazurin assay. After a 24 h incubation the TTR or vehicle were removed and the cells washed once with HBSS. Trypsin (0.125%) was used to detach the cells from the wells and to remove extracellular TTR associated with the cells. The collected cells were then washed twice with HBSS and lysed in RIPA-PI buffer as above. Analysis and quantification of the samples were performed by Western blot as above. Two biological duplicates were used per condition. The experiment was repeated three times. Average and standard deviations from one representative experiment are shown.

### Superoxide detection using DHE (dihydroethidium)

AC16 cells were seeded in 96-well plates (4000 cells/well) in Opti-MEM seeding medium. The following day the cells were treated with V122I TTR, T119M TTR, BSA (8 μM), Antimycin A (50 μM) or HBBS (control) for 24 h. The solutions were then removed and the cells loaded with 20 μM DHE (50 μl/well, Invitrogen) in HBSS for 20 min at 37°C. DHE is freely permeable to the cells and it is oxidized by superoxide to 2-hydroxy ethidium and ethidium [20]. These compounds bind to nucleic acids and emit a strong measurable red fluorescence [20]. Excess DHE was removed and the cells were washed once with HBSS (50 μl/well) and a final volume of 50 μl HBSS was added to each well. Fluorescence intensity was measured 24 h later using a multi-well spectrofluorimeter (Exc/Em 490/605 nm). Blanks consisted of AC16 cells treated with HBSS, not loaded with DHE but otherwise treated identically to the rest of the samples. Average and S.E.M. of six replicate values were calculated and plotted. Two tailed *t* tests were performed using GraphPrism (GraphPad) to determine the significance of differences between the treatments and HBSS (control). The experiment was repeated twice. Representative data from one experiment are shown.

### Caspase 3/7 activation

AC16 cells were seeded in 15 cm dishes at a density of 70000 cells/plate in Opti-MEM seeding medium and incubated at 37°C overnight. TTR (16 μM) or HBSS control were added and the cells incubated for 6 h at 37°C. After the TTR and HBSS were removed, the cells were washed with cold HBSS and collected in 15 ml tubes with the help of a cell scraper. The cells were washed three more times with cold HBSS followed by spin at 500 ***g*** and removal of buffer. A total of 400 μl of lysis buffer (50 mM Hepes, pH 7.4/1% Igepal CA-630, 1% Triton X-100 and 5 mM DTT) were added and the cells were subjected to one cycle of freeze/thaw followed by sonication on ice (1 min, amplitude 20, 3 s pulse with 1 s rest). The lysates were spun at 1500 ***g*** for 5 min at 4°C to remove the debris and nuclei. Twenty microliters of cell lysates were added to 100 μl of Caspase 3/7 substrate (Ac-DEVD-AFC, Enzo Biosciences) solubilized in 20 mM Hepes, pH 7.4/ 0.1% Igepal CA-630/ 0.1% Triton X-100, 2 mM EDTA, 5 mM DTT, in black 96-well plates (Corning). The lysates were then incubated at 37°C for 22 h and the fluorescence generated by cleavage of the substrate by caspases 3/7 was measured on a Tecan Safire2 (Exc/Em 405/500 nm with 5 nm bandwidth). The experiment was repeated twice. Data shown are the average of six replicates from one representative experiment and error bars are S.E.M.

## RESULTS

### Generation of a fluorescent TTR probe

In order to study the modes of interaction of TTR with target cells we generated fluorescent labelled V122I TTR variants. We used Oregon Green 488 maleimide and thiol chemistry to label the V122I TTR single cysteine in position 10 (Cys^10^). We also prepared two double mutant TTR variants in the V122I background to place a single cysteine at the C-terminus of the polypeptide chain, C10A/V122I/P125C TTR and C10A/V122I/E127C TTR. These two variants were then labelled with Oregon Green 488 maleimide and with Alexa Fluor 488-C5-maleimide. For our purposes, the ideal fluorescent V122I TTR variant must have the same aggregation propensity and the same cytotoxic potential as native V122I TTR.

### Aggregation properties of fluorescent labelled V122I TTR variants

**Figure 1:**
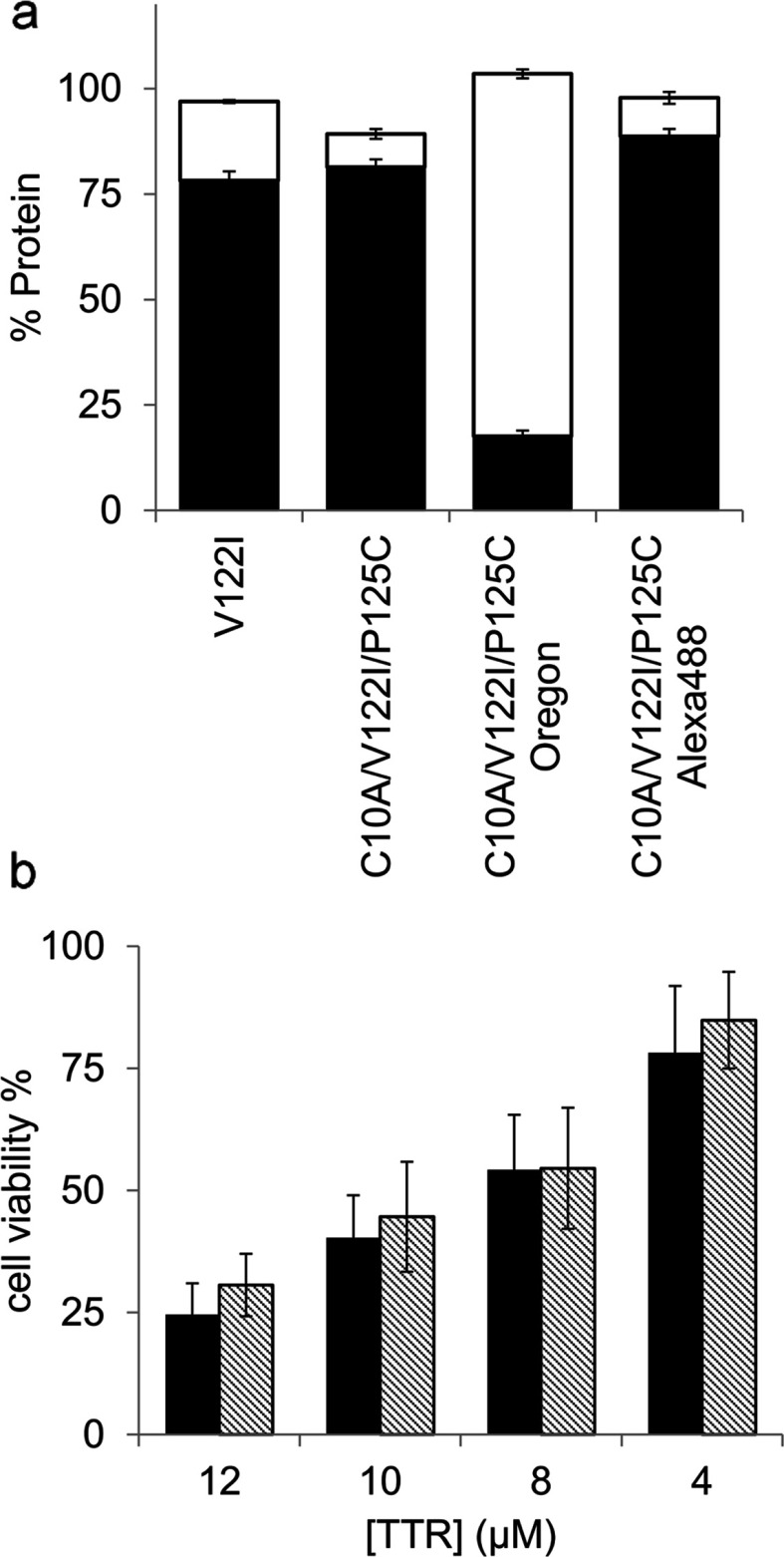
Aggregation capacity and cytotoxic potential of fluorescent-labelled V122I TTR variants (**a**) Percentage of aggregated (black bars) and soluble (white bars) protein for several V122I TTR variants after 3 days incubation at pH 4.4 and 37°C, with respect to total soluble protein at time zero. (**b**) Percentage of metabolic activity of the AC16 human cardiomyocyte cell line treated with V122I (black bars) or f-V122I (C10A/V122I/P125C-Alexa Fluor 488) TTRs, with respect to vehicle-treated cells.

TTR aggregation and fibril formation can be induced *in vitro* under mildly acidic conditions [5,21]. These conditions allow studying protein stability at convenient laboratory time scales. V122I TTR, the fluorescent labelled TTR isoforms and their unlabelled counterparts were incubated at pH 4.4 (the pH of maximum aggregation of V122I TTR) for 3 days at 37°C without agitation. In our experience, the previously described turbidimetric methods for the quantification of TTR amyloidogenesis do not accurately measure the aggregated protein, especially when different TTR variants are evaluated [5,18]; thus, we determined the extent of TTR aggregation by quantifying the percentage of protein that precipitates with respect to the total soluble protein at time zero (Figure 1a, black bars) [18]. We also measured the percentage of protein found in the supernates after the aggregated protein had been precipitated, with respect to total soluble protein at time zero (Figure 1a, white bars). For simplicity, we show the data corresponding to the mutant C10A/V122I/P125C TTR unlabelled, and labelled with Oregon Green 488 and Alexa Fluor 488, compared with native V122I TTR. The recovery yields of the aggregation reactions (precipitate+supernate) were near 100% for all the proteins tested. The unlabelled C10A/V122I/P125C TTR and the C10A/V122I/P125C-Alexa Fluor 488-labelled TTR variants have the same capacity for aggregation as native V122I TTR (Figure 1a, black bars). The V122I TTR variants labelled with Oregon Green 488 both, at the N- or C-terminus, had lower fibril formation capacity than V122I TTR (results shown for C10A/V122I/P125C-Oregon Green 488 variant).

### C10A/V122I/P125C-Alexa Fluor 488 TTR has the same cytotoxic potential as unlabelled V122I TTR

We chose the TTR variant labelled with Alexa Fluor 488 at position 125, C10A/V122I/P125C-Alexa Fluor 488 (named henceforth f-V122I TTR), to assess its cytotoxic potential against human cardiomyocytes. We have previously demonstrated that amyloidogenic TTR variants that deposit in the heart *in vivo* (i.e. V122I TTR, V30M TTR, V20I TTR and L111M TTR) are cytotoxic to the human cardiac cell line AC16, whereas the stable and non-amyloidogenic T119M TTR variant is not [13]. AC16 cells were treated with several concentrations of V122I TTR or f-V122I TTR for 24 h. Cell metabolic activity was measured by resazurin reduction assay and compared with cells treated with vehicle only. The data show that f-V122I has the same cytotoxic capacity as native V122I TTR (Figure 1b).

In summary, f-V122I TTR has the same aggregation and fibril formation propensity, and cytotoxic activity as native V122I TTR. Thus we used the fluorescent protein for our studies.

### The interaction of f-V122I TTR with human cardiac cells is saturable

**Figure 2:**
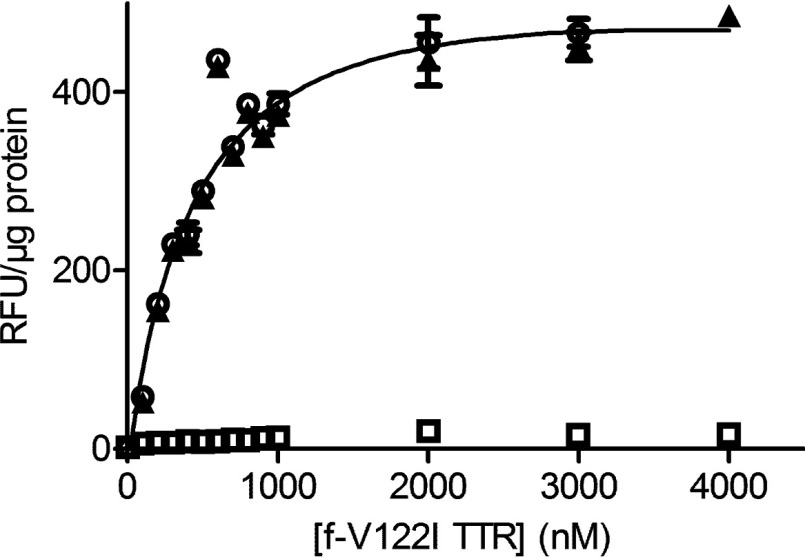
The interaction of f-V122I TTR with AC16 cells is saturable AC16 cells were incubated with increasing concentrations of f-V122I TTR or f-V122I TTR with 100× molar excess of unlabelled V122I to measure NSB. f-V122I TTR associated with the cells was measured by fluorescence intensity. These data were normalized for total protein content in the cell lysates measured by BCA assay. Total binding is denoted by open circles, NSB by open squares and specific binding (i.e. total-NSB) by black triangles. Specific binding data were fitted by a one site binding saturation curve (black line).

f-V122I TTR solutions at concentrations ranging from 100 to 4000 nM were incubated with the human cardiomyocyte cell line AC16 for 3 h at 4°C; the same concentrations of f-V122I with 100× molar excess of unlabelled V122I TTR were incubated with the cells in parallel to assess NSB. The cells were then thoroughly washed and lysed. The amount of f-V122I TTR associated with the cells was measured by fluorescence intensity and the data were normalized for total protein content in the samples. Plotted in Figure 2 are the total f-V122I TTR associated with the cells (total binding), NSB, and f-V122I TTR specific binding (total binding–NSB). The results show that the interaction of f-V122I TTR with the cells is saturable. The data best fit a one site binding saturation curve with an average K_D_ of 665±183 nM (four independent experiments, range 420–860 nM).

### Amyloidogenic f-V122I TTR competes with native V122I TTR, but not with the non-amyloidogenic T119M TTR or kinetically stabilized V122I TTR

**Figure 3:**
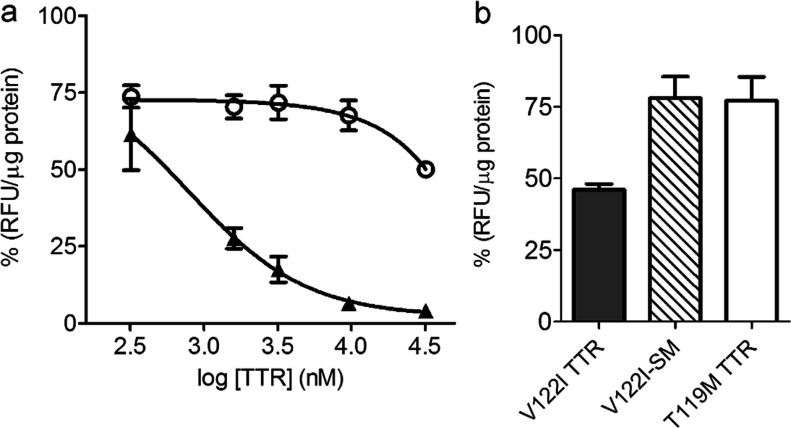
Native V122I TTR competes with f-V122I TTR for the same interaction sites in human cardiomyocytes (**a**) AC16 cells were co-incubated with f-V122I TTR (320 nM) and increasing concentrations of amyloidogenic V122I TTR or non-amyloidogenic T119M TTR for 3 h at 4°C. The fluorescence associated with the cells was measured and normalized for total protein content. The data were normalized by the fluorescence from the samples incubated with f-V122I TTR only (100%). Filled triangles, V122I TTR; open circles, T119M TTR. (**b**) AC16 cells were co-incubated with f-V122I (600 nM) and equimolar amounts of unlabelled V122I TTR, T119M TTR, V122I stabilized with a small molecule (V122I-SM) or HBSS. Fluorescence associated with the cells was measured, normalized by total protein content and plotted as percentage with respect to the fluorescence of cells that had been incubated with f-V122I and HBSS only. The data show that similarly to T119M TTR, V122I-SM is unable to efficiently displace cell-associated f-V122I, whereas under the same conditions V122I TTR displaces more than 50% of f-V122I TTR.

The differential cytotoxicity behaviour displayed by V122I TTR and T119M TTR could be related to how these two variants interact with the target cells. To test this notion, AC16 cells were incubated with f-V122I TTR (320 nM) and increasing concentrations of amyloidogenic V122I TTR or non-amyloidogenic T119M TTR for 3 h at 4°C, a length of time sufficient to reach steady state. The cells were then washed and the fluorescence associated with the cells measured. The fluorescence intensity data were normalized by total protein in the cell lysates and are represented as percentage of fluorescence associated with the cells with respect to samples that were incubated with f-V122I TTR only (Figure 3a). V122I TTR competes with f-V122I TTR for cell binding with an average IC_50_ of 638±182 nM (range 391–872 nM, six independent experiments) which corresponds to a dissociation constant for V122I, Ki of 429±122 nM (range 263–587 nM). For T119M TTR there was an initial displacement of about 20% f-V122I TTR associated with the cells which was maintained over the concentration range of 320–16,000 nM. Only at 100× molar excess of T119M TTR (32 μM) with respect to f-V122I TTR was there an additional decrease in the amount of fluorescence associated with the cells. These data demonstrate that T119M TTR is very inefficient at displacing f-V122I TTR from the cells and suggest that either f-V122I TTR and T119M TTR interact with the cells at different sites, or that the affinity of T119M TTR for the cell binding site is much lower than that of f-V122I TTR and by inference to that of V122I TTR.

Similar results were found in an experimental setting in which the unlabelled proteins (V122I and T119M TTR) were first pre-incubated with the cells for 1 h at 4°C followed by incubation with increasing concentrations of f-V122I TTR. In this case, the fluorescence associated with cells pre-incubated with T119M TTR was very similar to that of cells pre-incubated with vehicle only (HBSS) at all f-V122I TTR concentrations tested, whereas it was significantly lower for cells pre-incubated with V122I TTR (Supplementary Figure S1).

Resveratrol and many of its structural analogs bind to and stabilize native tetrameric TTR preventing its aggregation and fibril formation *in vitro*, and inhibiting V122I TTR-induced cytotoxicity in the human cardiomyocyte cell line AC16 [13,16]. We stabilized V122I TTR with one such resveratrol analogue that binds covalently to Lys^15^ located in the TTR T_4_ binding pocket (compound 4 in reference [16], henceforth referred to as SM) and measured its capacity to compete with f-V122I TTR. Usage of a covalent binder allowed us to obtain pure SM-modified V122I TTR free of SM excess (see Experimental section). The data show that at 1:1 molar equivalents of f-V122I TTR: unlabelled TTR (600 nM), V122I TTR displaces more than 50% of f-V122I fluorescence. Under the same conditions, T119M TTR and SM-stabilized V122I TTR (V122I-SM) can only displace 22–23% of f-V122I fluorescence from the cells (Figure 3b). Thus, stabilizing V122I TTR with SM decreases its capacity to displace f-V122I TTR from the cells to the same degree as the highly stable T119M TTR variant.

### Amyloidogenic and non-amyloidogenic TTR variants interact differently with the human cardiomyocytes

**Figure 4:**
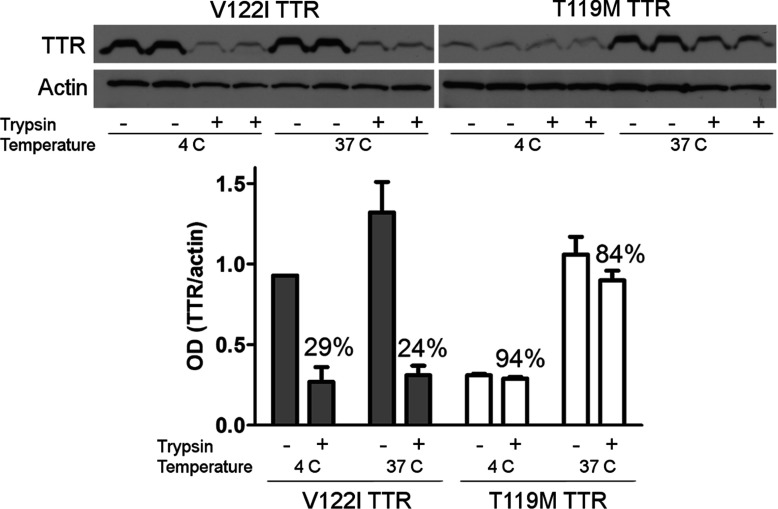
Differential interaction of the amyloidogenic V122I TTR and the non-amyloidogenic T119M TTR with the human cardiac cell line AC16 AC16 cells were treated with trypsin (or HBSS as control) to remove membrane-associated proteins. V122I and T119M TTR were then incubated with the cells for 4 h at 4°C or 37°C. Cell lysates were prepared and analysed by SDS—PAGE (15% gel) and Western blot using an anti-TTR and anti-actin antibodies. The lower panel shows densitometry analysis (absorbance) of total cell-associated TTR normalized to actin. Error bars represent S.D. of the biological duplicates. The percentage of TTR/actin ratio in cells pre-treated with trypsin (trypsin +) with respect to control cells (trypsin −) is shown.

To further define how the amyloidogenic (V122I) and non-amyloidogenic (T119M) proteins interacted with the cells we incubated the human cardiomyocytes with trypsin (or HBSS as control) to remove the exposed membrane proteins. The process was followed by incubation with amyloidogenic V122I TTR or non-amyloidogenic T119M TTR at 4°C (to prevent energy-dependent processes) and at 37°C for 4 h. Total TTR associated with the cells was measured and quantified by Western blot as detailed in the Experimental section. The data show that trypsin pre-treatment resulted in a 70–75% reduction of the total V122I TTR associated with the cells, compared to no trypsin-treated (control) samples, both at 4°C and at 37°C (Figure 4). The same treatment did not affect the interaction of T119M with the cells. Thus, the mode of interaction of V122I TTR and T119M TTR with the cells is different. The data also indicate that a specific membrane protein(s) might be involved in the interaction of the amyloidogenic V122I TTR with the cells but not in the interaction of the non-toxic T119M TTR variant.

### Amyloidogenic V122I TTR is mainly associated with the cell membrane, whereas non-amyloidogenic T119M TTR is mainly internalized

**Figure 5:**
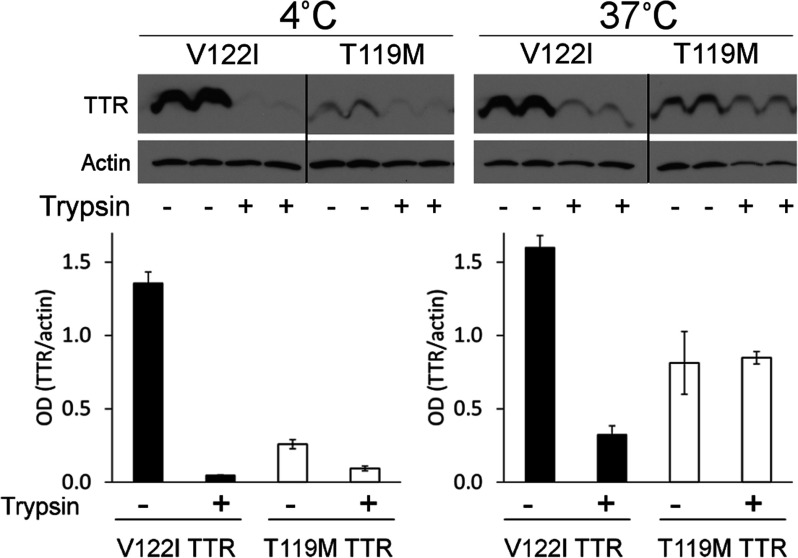
Aberrant interaction of V122I TTR with human cardiomyocytes AC16 cells were incubated for 3 h with amyloidogenic V122I TTR or non-amyloidogenic T119M TTR (4 μM) at 4 or 37°C. Half of the experimental samples were then incubated with trypsin to remove the extracellular cell membrane-associated TTR. Cell lysates were analysed by SDS–PAGE and Western blot using an anti-TTR antibody and actin (upper panels). Band intensities were quantified by densitometry (absorbance) and plotted in the lower panels as TTR/actin ratio.

To further investigate the modes of interaction of TTR with the human cardiomyocytes, cytotoxic V122I TTR and non-cytotoxic T119M TTR were first incubated with the AC16 cells for 3 h at 4°C or 37°C. After removing the TTR solutions, half of the samples were treated with trypsin to remove the TTR associated with the cell membrane. Cell lysates were prepared and analysed by SDS–PAGE and Western blot using a polyclonal anti-TTR antibody (Figure 5). Control experiments using recombinant V122I TTR and T119M TTR indicate that the polyclonal antibody used for the Western blot experiments has similar reactivity for the two proteins (results not shown). The data show that at 4°C there is at least five times more V122I TTR than T119M TTR associated with the cells. At 4°C, the samples that were treated with trypsin after incubation with V122I or T119M TTR have a much lower TTR signal, consistent with the notion that at 4°C the TTR interacting with the cells is extracellular. At 37°C, a temperature that allows energy-dependent TTR internalization, the total amount of T119M TTR interacting with the cells increases compared with the incubations performed at 4°C. Treatment of the cells with trypsin after incubation with TTR at 37°C resulted in an 80% decrease of the V122I TTR associated with cells, but no change in the cells treated with T119M TTR. These results indicate that most of the V122I TTR associated with the cells remains extracellular, whereas most of the T119M TTR associated with the cells is intracellular. Using glycine buffer (0.2 M glycine/150 mM NaCl, pH 2.0), another classical protocol to remove membrane-associated proteins that does not depend on a enzymatic activity [23], resulted in the removal of 95–35% of V122I TTR associated with the cells, depending on the experimental conditions (results not shown). Since the trypsin protocol gave more consistent results from experiment to experiment, we subsequently focused only on this method to remove membrane-associated proteins.

### Both, the amyloidogenic V122I TTR and non-amyloidogenic T119M TTR are directed to the proteasome/lysosome for degradation

**Figure 6:**
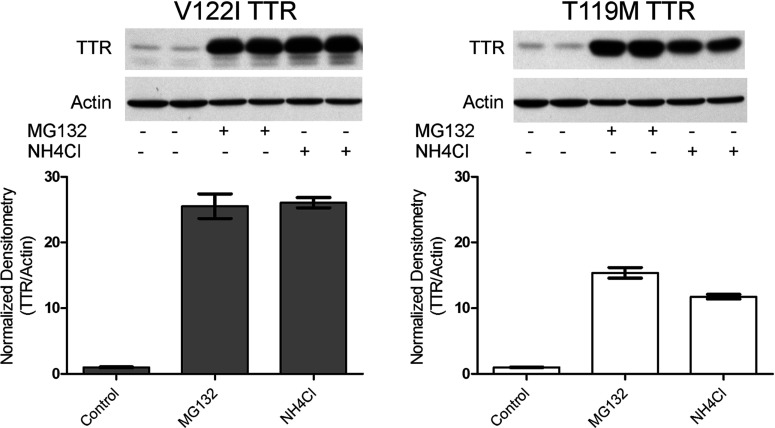
V122I TTR and T119M TTR accumulate in the presence of proteasomal (MG132) and lysosomal (NH_4_Cl) activity inhibitors AC16 cells were pre-incubated for 30 min with 0.5 μM MG132, 10 mM NH_4_Cl or vehicle only (control). V122I TTR and T119M TTR were then added at a final concentration of 4 μM and the cells were incubated for 24 h at 37°C. Cell lysates were prepared after removing cell membrane-associated TTR with trypsin, and analysed by SDS–PAGE and Western blot developed with anti-TTR antibody and anti-actin antibody. Data are presented as TTR/actin ratio for each treatment and normalized to the average ratio values obtained in cells incubated with the vehicle only.

Most of the non-cytotoxic T119M TTR and a small fraction of the amyloidogenic V122I TTR interacting with the AC16 human cardiomyocytes are internalized. To determine the fate of the internalized TTR, we treated AC16 cells with proteasomal (0.5 μM MG132) and lysosomal (10 mM NH_4_Cl) activity inhibitors for 24 h. We chose working concentrations that were minimally toxic as measured by resazurin assay (results not shown). Total TTR associated with the cells was quantified using SDS–PAGE and Western blot analysis (Figure 6). Both V122I and T119M TTR accumulate inside the cells in the presence of lysosomal and proteasomal inhibitors suggesting that once TTR is internalized, whether it is amyloidogenic or not, it follows the same degradation pathway. These observations were not limited to MG132 and NH_4_Cl since other inhibitors of the proteasomal (bortezomib) and lysosomal (choroquine) activities also resulted in accumulation of both V122I TTR and T119M TTR inside the cells (results not shown).

### V122I TTR cytotoxicity is dependent on membrane cholesterol content

**Figure 7:**
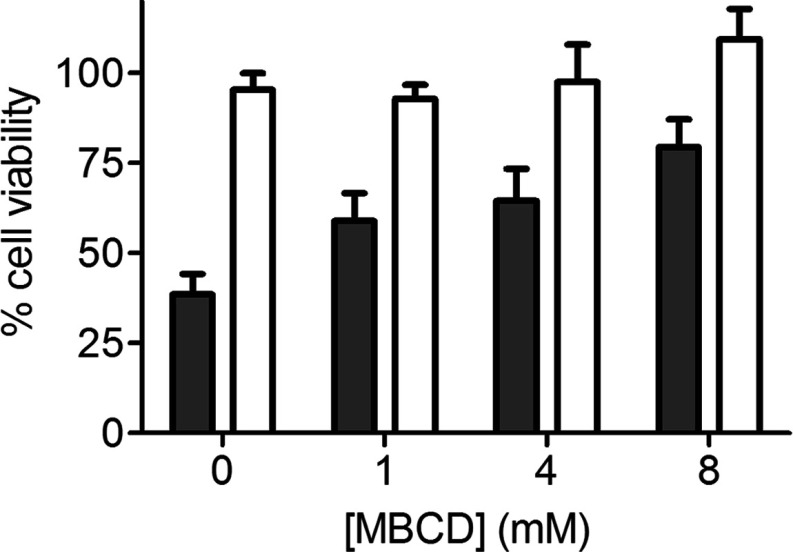
MBCD decreases V122I TTR-induced cytotoxicity in human cardiomyocytes in a dose-responsive manner AC16 cells were incubated with several concentrations of MBCD (0 to 8 mM) for 15 min followed by incubation with amyloidogenic V122I TTR (grey bars) or non-amyloidogenic T119M TTR (white bars) at 8 μM. After 24 h incubation at 37°C, the percentage of cell viability with respect to vehicle-treated cells was measured by resazurin assay.

In other protein misfolding systems, it has been shown that membrane cholesterol content has an impact in the interaction of the proteins with the cell membrane and in their subsequent cytotoxicity [24–26]. To determine whether this was the case in our system, we treated the AC16 human cardiomyocytes with several concentrations of MBCD to decrease membrane cholesterol, followed by incubation for 24 h with amyloidogenic V122I TTR or the non-amyloidogenic T119M TTR. Cell metabolic activity was measured by resazurin reduction as described in [13]. Consistent with our previous observations, V122I TTR but not T119M TTR decreased cell metabolic activity (Figure 7). MBCD decreased V122I TTR induced cytotoxicity in a dose-responsive manner but had no significant effect on T119M TTR treated cells. Under these conditions MBCD treatment alone (without TTR) did not significantly decrease cell viability with respect to cells treated with vehicle only (results not shown). These results suggest that membrane cholesterol homoeostasis may have a significant role in the V122I TTR cytotoxic process.

### MBCD does not prevent V122I TTR aggregation

Many small molecules bind in the T_4_ binding pocket of TTR and prevent its aggregation and amyloidogenesis cascade by stabilizing the native tetramer [27–29]. They also prevent amyloidogenic TTR induced cytotoxicity [12,13,16,22,30–32]. To determine whether the decrease in V122I TTR-induced cytotoxicity by MBCD could be caused by a putative interaction with the T_4_ binding pocket of TTR resulting in native tetramer stabilization, we performed standard aggregation assays using recombinant V122I TTR in the presence/absence of MBCD [18] (Supplementary Figure S2). Resveratrol, a well-known TTR stabilizer was used as a positive control. The data show that in contrast to resveratrol, MBCD does not prevent V122I TTR aggregation; thus, its effect on decreasing V122I TTR-induced cytotoxicity is not due to tetramer stabilization, but most likely, to its capacity to deplete membrane cholesterol.

### MBCD removes membrane cholesterol from human cardiomyocytes and modulates the interaction and trafficking of TTR with the cells

Treatment of AC16 cells with 8 mM MBCD for 0.25–24 h resulted in a time-dependent decrease of membrane cholesterol that ranged from 30 to 78% as measured by Amplex Red cholesterol assay (Supplementary Figure S3). In a 4 h incubation period 54% of the cholesterol was removed with less decrease in cell viability than after overnight treatment. Hence, we used those conditions to evaluate the association of amyloidogenic V122I TTR and non-amyloidogenic T119M TTR with AC16 cells.

**Figure 8:**
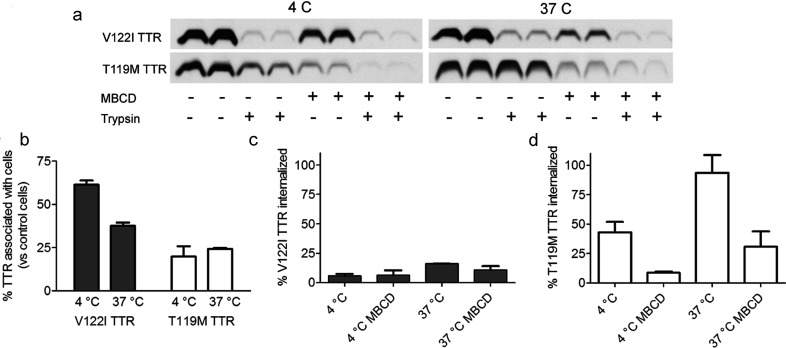
MBCD interferes with the interaction of TTR with human cardiac cells AC16 cells were incubated with amyloidogenic (V122I) or non-amyloidogenic (T119M) TTR in the presence or absence of 8 mM MBCD for 4 h at 4 or 37°C. Afterwards, half of the samples were treated with trypsin to remove membrane-associated TTR. Cell lysates were prepared and analysed by SDS–PAGE and Western blot and developed with an anti-TTR antibody (**a**). TTR band density was measured and the percentage of TTR associated with the MBCD-treated cells was calculated with respect to vehicle (DMSO)-treated cells (**b**). The percentage of internalized V122I TTR (**c**) and T119M TTR (**d**) was calculated from the density values of cells treated with trypsin with respect to cells not treated with trypsin.

Cell lysates of samples incubated with or without trypsin after the MBCD and TTR treatments were prepared and analysed to assess the total and internalized protein as above (Figures 8a–8d). In these experiments, MBCD affected the amounts of actin, tubulin and total protein in the cell lysates without affecting cell number; thus, we could not use these proteins to normalize the Western blots. However, in all our previous experiments, we noticed that normalizing the TTR bands to actin did not influence the biological observations in a significant manner (see below). For these reasons, together with the fact that we ran independent biological duplicates in each experiment to account for sample handling variation, we quantified the TTR bands without an internal normalization standard.

There is a moderate to high decrease of the V122I TTR associated with the cells in the presence of MBCD (Figure 8b) with 61±2.5 and 37±1.9% of V122I TTR remaining at 4 and 37°C, respectively, compared with V122I TTR-treated cells (no MBCD). The effects of MBCD on the association of T119M TTR with the cells are more dramatic, with only 20±6 and 24±0.6% remaining at 4 and 37°C, respectively, compared with T119M TTR-treated cells (no MBCD).

The effect of MBCD on TTR internalization was calculated as a percentage of the densitometry values of the samples of interest (trypsin treated samples) to their corresponding controls (non-trypsin treated samples) (Figure 8 panels (c) for V122I and (d) for T119M). The data are consistent with our prior observations (Figure 5) in that the V122I TTR associated with the cells is essentially extracellular: there is only 5.9±1.7 and 16.1±0.2% V122I TTR at 4 and 37°C, respectively, associated with the cells in the samples treated with trypsin (intracellular), with respect to non-trypsin-treated cells (total TTR). For T119M TTR there is 43.0±8.9 and 93.3±15.1% at 4 and at 37°C, respectively, that are associated with the cells treated with trypsin (intracellular) with respect to non-trypsin-treated cells (total TTR) (Figure 8d). These data also validate the notion that the absence of an actin standard for the TTR densitometry does not change the biological findings (see above).

With respect to the MBCD effect on TTR internalization the data show no significant changes on internalized V122I TTR at 4°C (5.9±1.7 versus 6.4±4.2% without versus with MBCD, respectively) and a slight decrease at 37°C (16.1±0.2 versus 10.8±3.3% without versus with MBCD) (Figure 8c). The effects of MBCD on V122I TTR internalization are not pronounced because the amount of V122I TTR internalized is small. Conversely, there is a large decrease of internalized T119M TTR from 43.0±8.9 to 8.7±1.1%, without and with MBCD, respectively, at 4°C, and from 93.6±15.1 to 30.8±13.1% without and with MBCD, respectively, at 37°C (Figure 8d). These findings have two implications: first, that membrane cholesterol plays a role in the interaction of TTR (amyloidogenic and non-amyloidogenic) with the cells; and second, that the observed T119M TTR internalization is highly dependent of membrane cholesterol presence.

### V122I TTR induces superoxide species formation and caspase 3/7 activation in human cardiomyocytes

**Figure 9:**
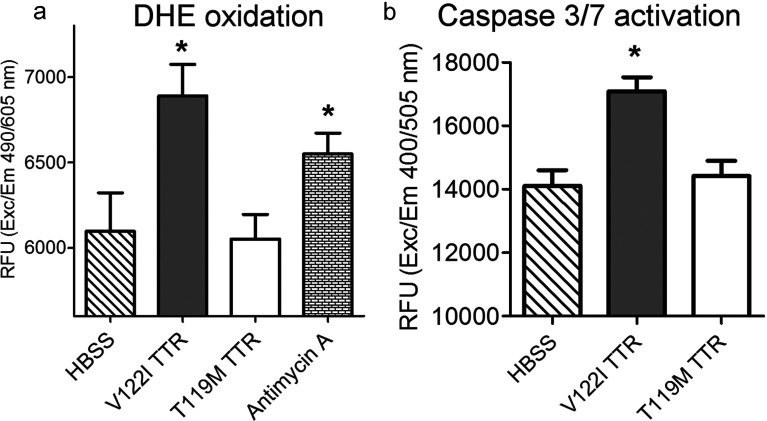
Amyloidogenic V122I but not non-amyloidogenic T119M TTR induces the generation of superoxide species and caspase 3/7 activation in human cardiomyocytes (**a**) AC16 cells were treated with V122I TTR, T119M TTR, Antimycin A (positive control) or HBSS (vehicle control) for 24 h. The insults were then removed and the cells loaded with the superoxide-specific reagent DHE as detailed in the experimental section. Red fluorescence produced by DHE oxidation is shown. (**b**) AC16 cells were treated with V122I TTR, T119M TTR or, HBSS for 6 h at 37°C. Cell lysates were prepared as detailed in the experimental section and were added to Ac-DEVD-AFC caspase 3/7 substrate. Fluorescence intensity generated by cleavage of the substrate was measured. Asterisks in both panels indicate statistical significance with *P*<0.05 of experimental samples compared to HBSS (vehicle control) treated cells (unpaired *t* test).

There is a body of literature linking protein misfolding and amyloidosis, including TTR amyloidosis, to the generation of ROS and apoptotic cell death [33–35]. Thus, we tested whether such mechanisms were also involved in TTR-induced cytotoxicity in the human cardiac AC16 cells. We have shown that amyloidogenic V122I TTR but not the non-amyloidogenic T119M TTR reduces cell viability in human cardiomyocytes as measured by resazurin reduction (Figure 7) [13]. To assess whether ROS were generated under conditions in which we observe a decrease in cell viability, we measured DHE oxidation. AC16 cells were exposed to V122I TTR, T119M TTR or HBSS for 24 h. Antimycin A, an inhibitor of complex III of the electron transport chain which results in an accumulation of superoxide, was used as positive control [36]. After the insults were removed, the cells were treated with DHE as detailed in the experimental section. The data demonstrate that DHE oxidation increases in V122I TTR-treated cells relative to control cells (*P*<0.05, *t* test) (Figure 9a). Non-amyloidogenic T119M TTR treated cells had a fluorescent intensity not significantly different from that of the control cells (HBSS), indicating that the effect is specific for amyloidogenic TTR. Similar results were found when the DHE dye was loaded into the cells before adding the proteotoxic insults (results not shown). Analogous experiments were performed to determine whether V122I TTR induced generation of peroxide, peroxynitrite, hydroxyl radical or nitric oxide using the dye CM-H_2_-DCFDA [5-(and-6-)chloromethyl-2′,7′-dichlorodihydrofluorescein diacetate, acetyl ester—] as substrate [37]. Several conditions covering a wide range of cell densities, V122I TTR concentrations and incubation times did not result in a significant increase in DCF fluorescence compared with HBSS-treated cells (results not shown), suggesting that these specific ROS were not generated by V122I TTR interacting with the cells.

To assess whether V122I TTR induced caspase 3/7 activation, a marker of apoptosis or programmed cell death that is known to be activated in TTR amyloidosis patients [35] and in other TTR tissue culture models [12,15,38], we used cell lysates from V122I, T119M and HBSS-treated cells to cleave the substrate Ac-DEVD-AFC as detailed in the experimental section. The data show that the amyloidogenic V122I but not the non-amyloidogenic T119M TTR cleaves the caspase 3/7 substrate resulting in a significant increase in AFC fluorescence with respect to control cells. Overall our results are compatible with apoptosis being involved in V122I TTR-induced cell damage (Figure 9b).

## DISCUSSION

There is an accumulating body of data coming from human biopsies, human TTR transgenic mouse models and tissue culture systems, indicating that in the TTR amyloidoses, tissue dysfunction precedes TTR fibril deposition [8,11–13]. In order to examine the mechanisms responsible for the observed non-fibrillar tissue damage we used our previously developed human cardiomyocyte tissue culture system, a model for FAC, to define how amyloidogenic and cytotoxic V122I TTR and non-amyloidogenic and non-cytotoxic T119M TTR variants interact with the cardiac cells. The amyloidogenic V122I TTR variant is the most common mutation worldwide, whose deposition in the heart results in one of the manifestations of FAC [3]. The naturally-occurring stable and non-amyloidogenic T119M TTR variant has been shown to prevent or delay TTR deposition in carriers of the otherwise amyloidogenic V30M TTR (V30M) when is expressed in *trans* (*i.e.* in different alleles) [39]. Both V122I and T119M TTR variants have been extensively characterized *in vitro*: the amyloidogenicity of V122I TTR is due to the low kinetic stability of its native tetramer hence, the rate of disassembly into its corresponding misfolding-prone monomers is accelerated compared to that of WT TTR [40]. Conversely, T119M TTR is a kinetically stable variant whose tetramer-monomer transition is energetically very unfavourable, thus it remains as a tight homotetramer in solution [41]. The presence of one single T119M TTR subunit in a tetramer otherwise composed of WT or V30M TTR is enough to kinetically stabilize the resulting quaternary structure [41].

Our approach is unique in that it compares directly two soluble TTR variants with extremely different amyloidogenic and cytotoxic potential on cells that are relevant to TTR cardiac amyloidosis. This strategy has allowed us to clearly differentiate the behaviour of the amyloidogenic and non-amyloidogenic TTR variants. Other studies of the interaction of TTR with cells either are confined to WT TTR in the context of its catabolism, often limited to hepatic targets, its T_4_ transport properties, or assume that TTR is pre-aggregated before it interacts with cells or artificial membranes. This assumption requires the unlikely event that these large, insoluble TTR aggregates circulate in plasma.

For our studies we created a fluorescent labelled TTR variant with sequence C10A/V122I/P125C to which Alexa Fluor 488 dye was appended through maleimide chemistry, yielding a variant with the same aggregation and amyloid fibril formation capacity as native V122I TTR (Figure 1). Under mildly acidic conditions mainly amorphous aggregates and some protofibrils, but not mature amyloid fibrils, are obtained [42]. The labelled protein, which we termed f-V122I TTR, has also the same cytotoxic potential as native V122I TTR, thus it is ideally suited for our intended studies. Labelling TTR with a fluorescent tag rather than a previously used radioactive probe [43,44], has proven to be sensitive enough for the study of the interaction of TTR with the cells.

The association of f-V122I TTR with the human cardiomyocyte cell line AC16 at 4°C shows a hyperbolic dependence with the f-V122I TTR concentration with an estimated dissociation constant *K_D_*=665 nM (Figure 2). Unlabelled amyloidogenic V122I TTR could displace f-V122I TTR from the cells (Figure 3 and Supplementary Figure S1). Conversely, the non-amyloidogenic T119M TTR was not able to compete with f-V122I TTR. Furthermore, a kinetically stabilized V122I TTR (V122I-SM) by a small molecule that binds covalently in the TTR T_4_ binding pocket, behaves like the kinetically stable T119M TTR in that it has low capacity to compete with f-V122I TTR for cell surface binding (Figure 3b). These observations attest the specificity of the amyloidogenic V122I TTR for surface cell binding. It is noteworthy that neither of the two TTR mutation sites of the TTR variants under study (Ile^122^ or Met^119^) are located in an exposed region of the tetramer; both are located at the TTR dimer-dimer interface. Thus, the differential interaction of V122I TTR and T119M TTR with the cells is most likely not related to the mutations themselves (i.e. one of the mutated amino acids interacting directly with some element on the cell membrane); rather, it may be due to conformational differences between the two quaternary structures (see below).

The dependence of V122I TTR on membrane protein(s) for interaction with the human cardiac cells, as well as the hyperbolic saturability behaviour observed for f-V122I TTR is reminiscent of a receptor-mediated specific interaction. TTR receptors have been invoked for the internalization of soluble WT TTR in the context of normal catabolism in hepatocytes, and T_4_ transport in astrocytoma, chicken oocytes and ependymoma cells, although none has been identified [43,45–47]. In hepatoma cell lines and in mouse primary hepatocytes, WT TTR internalization was described to be a receptor-mediated process [44]. In that work it was shown that RAP (receptor-associated protein) prevented TTR internalization and it was concluded that and unidentified RAP mediated WT TTR internalization. The affinity of WT TTR for the putative receptor was in the low nanomolar range. It was indirectly shown that TTR variants with lower stability (more amyloidogenic) had lower levels of internalization than the non-amyloidogenic T119M TTR in the hepatocytes [44]. In a different tissue culture system, megalin, a scavenger receptor of the LDL (low-density lipoprotein) family, has been described to play a role in TTR catabolism in the kidney, with higher levels of internalization observed for amyloidogenic TTR variants than for non-amyloidogenic TTR [48]. The affinity of TTR for this receptor was estimated to be ~500 nM. Our data together with the previously published studies suggest that the interaction of TTR with cells is conformation-dependent and tissue-specific. We propose that different cell types may interact with, and process different TTR quaternary species in distinct manners, explaining the observed variability in binding affinities, internalization efficiencies, and protein dependence of the interaction process among the different experimental systems.

Our data show that the amyloidogenic V122I TTR interacts differently with the human cardiomyocytes than does the non-amyloidogenic T119M TTR. The findings point directly to the possible cause of the early events in tissue damage: the deposition of the amyloidogenic V122I TTR on the membrane as opposed to the non-pathogenic internalization and protein degradation pathway characterized by the interaction of the non-amyloidogenic T119M TTR with the human cardiomyocytes (Figures 4 and 5). The small fraction of V122I protein that is internalized appears to follow the same path as the bulk of T119M, i.e., degradation in the lysosome (Figure 6). These observations suggest that it is the extracellular membrane-associated V122I TTR that might cause the observed decrease in cell viability, perhaps blocking the surface of the cell and interfering with normal protein/ion transport. An increase of cell membrane fluidity by TTR and a disruption of Ca^2+^ homoeostasis have been proposed as possible mechanisms of TTR-induced cytotoxicity [49,50], although these experiments were performed with artificial liposomes.

The extent of V122I TTR interaction with the cells and its cytotoxicity decreases in cells with reduced membrane cholesterol. Cholesterol has several functions: it gives permeability and modulates fluidity to the plasma membrane; it is involved in intracellular transport as part of vesicles; and it is a component of lipid rafts which cluster cell receptors and secondary messenger molecules [51]. Given that it appears that the cytotoxic process comes from the V122I TTR associated with the cell membrane, we hypothesize that the role of cholesterol on the TTR-induced cytotoxic cascade might be that of bringing together protein receptors and/or secondary messengers. Consistent with our data, Hou et al. showed that lipid bilayers with increasing mole fractions of cholesterol also resulted in increased affinity of the amyloidogenic L55P TTR variant for the lipid bilayers [52]. Previous studies using isolated cell membranes and/or synthetic liposomes indicate that pre-aggregated TTR has affinity for ionic phospholipids rather than proteins, as measured by surface plasmon resonance [50,52]. Whether these observations will also be true in whole cells has not yet been determined. Our experimental approach differs from the aforementioned studies in that TTR is not pre-aggregated at the outset of the experiments–as it is likely to be the case *in vivo*–and that we use intact cells rather than isolated membranes or synthetic bilayers to probe the interaction of the TTR variants with the cells.

The interaction and internalization of T119M TTR with the human cardiomyocytes is highly dependent on membrane cholesterol (Figure 8). The observation that T119M TTR does not require a surface protein to interact with the cells, together with the fact that removal of membrane cholesterol decreases its internalization, suggest that the internalization mechanism might be mediated by lipid rafts in a receptor-independent manner [53].

Our data raise the question of which specific TTR species are interacting with the cells? At this stage we can but speculate. Both recombinant T119M TTR and V122I TTR are purified as tetrameric proteins, and these are the species initially incubated with the cells. *In vitro*, TTR can dissociate and aggregate under mild acidic conditions, as well as in the presence of liposomes enriched with acidic phospholipids [54]. It has been suggested that the composition of the cell membrane might produce microdomains with low pH due to acidic phospholipids such as phosphatidyl serine [54]. If such microdomains exist on living cells, the interaction of tetrameric V122I TTR with the membrane could induce its disassembly and oligomerization, and trigger the aggregation and cytotoxic cascade. T119M TTR would remain tetrameric because of its high kinetic stability. pH measurements of the plasma membrane and the glycocalyx (the extracellular matrix surrounding the cells) in melanoma cells, indicate that their values are between 7.4 and 7.6, respectively, when the external pH is 7.2, and near 6.75 when the external pH is 6.5 [55]. In epithelial cells the pH values of the cell membrane are close to 7.2 with lower values obtained at the cell junctions (pH 6.2) [56]. If these pH values are similar to those in human cardiomyocytes, they do not appear to be sufficiently low to promote TTR dissociation and aggregation.

Another explanation for the differential interaction of amyloidogenic and non-amyloidogenic TTR variants with the cells is that the conformations of the V122I TTR and T119M TTR tetramers are different. This notion would be consistent with the two tetramers interacting differently with the cells, allowing T119M TTR to be rapidly internalized while V122I TTR would remain associated with the cell membrane in an unproductive and damaging manner. Such possibility is supported by data generated by us and others demonstrating that recombinant WT TTR purified at 4°C or at 37°C has different cytotoxic potential which might indicate that the protein may have at least two alternative tetrameric conformations with different stability [13,15].

A third possibility is the notion that there might be a small amount of V122I TTR in solution that is not tetrameric (monomer or small oligomers) due to the rapid dissociation of the tetramer which is characteristic of molecules with low kinetic stability. These monomers or oligomers could be efficiently captured by the cells and removed from solution. These species would re-form rapidly in solution and be adsorbed by the cells, accumulating on the membrane. In contrast, the high kinetic stability of T119M TTR precludes the formation of these species, and only tetrameric T119M TTR is likely to circulate and interact with the cells.

Once amyloidogenic V122I TTR interacts with the cells it reduces cell viability (Figure 7) and [13], resulting in the generation of superoxide species and caspase 3/7 activation (Figure 9), a phenomena not seen in cells treated with the non-amyloidogenic T119M TTR variant. Future studies will be directed at understanding the precise mechanism and sequence of events that result in these observations. Similarly, the use of primary cardiomyocytes will be useful to confirm the observations made in the AC16 cell line.

In summary, we have shown that amyloidogenic V122I TTR accumulates on the cell membrane of human cardiomyocytes and is not internalized and trafficked for degradation as is the non-amyloidogenic T119M TTR variant. The interaction of V122I TTR with the cells appears to depend on membrane protein(s) and is modulated by membrane cholesterol. The findings suggest a new avenue for therapeutic intervention which would involve preventing the pathogenic interaction of V122I TTR with the cells. We propose that strategies other than native tetramer stabilization that directly modulate the interaction of the amyloidogenic protein with the cell surface, can result in alternative or complementary treatment for the TTR amyloidoses. For example, mechanisms that increase the fraction of V122I TTR that is internalized and degraded by the cells could prove beneficial to prevent tissue damage; strategies directed to prevent the physical interaction of the amyloidogenic proteins with the cell membrane would also be beneficial. Understanding the early mechanisms of TTR non-fibrillar proteotoxicity will provide tools to devise prophylactic and intervention strategies to prevent and/or delay the onset of tissue damage. These approaches might be more broadly applicable to other amyloidoses such as light chain amyloidosis or AD (Alzheimer's disease).
